# Association between Physician Specialty and Risk of Prescribing Inappropriate Pill Splitting

**DOI:** 10.1371/journal.pone.0070113

**Published:** 2013-07-29

**Authors:** Chia-Yu Chou, Chia-Chen Hsu, Shu-Chiung Chiang, Chin-Chin Ho, Chia-Lin Chou, Min-Shan Wu, Yuh-Lih Chang, Han-Yi Tsai, Tzeng-Ji Chen, Yueh-Ching Chou

**Affiliations:** 1 Department of Critical Care Medicine, Taipei Veterans General Hospital, Taipei, Taiwan; 2 Department and Institute of Pharmacology, National Yang-Ming University, Taipei, Taiwan; 3 College of Pharmacy, Taipei Medical University, Taipei, Taiwan; 4 Department of Internal Medicine, School of Medicine, National Defense Medical Center, Taipei, Taiwan; 5 Institute of Biomedical Informatics, National Yang-Ming University, Taipei, Taiwan; 6 Department of Pharmacy, Taipei Veterans General Hospital, Taipei, Taiwan; 7 Institute of Hospital and Health Care Administration, National Yang-Ming University, Taipei, Taiwan; 8 Department of Family Medicine, Taipei Veterans General Hospital, Taipei, Taiwan; Johns Hopkins School of Public Health, United States of America

## Abstract

**Background:**

Prescription errors that occur due to the process of pill splitting are a common medication problem; however, available prescription information involving inappropriate pill splitting and its associated factors is lacking.

**Methods:**

We retrospectively evaluated a cohort of ambulatory prescriptions involving extended-release or enteric-coated formulations in a Taiwan medical center during a 5-month period in 2010. For this study, those pill splitting prescriptions involving special oral formulations were defined as inappropriate prescriptions. Information obtained included patient demographics, prescriber specialty and prescription details, which were assessed to identify factors associated with inappropriate pill splitting.

**Results:**

There were 1,252 inappropriate prescriptions identified in this cohort study, representing a prescription frequency for inappropriate pill splitting of 1.0% among 124,300 prescriptions with special oral formulations. Among 35 drugs with special oral formulations in our study, 20 different drugs (57.1%, 20/35) had ever been prescribed to split. Anti-diabetic agents, cardiovascular agents and central nervous system agents were the most common drug classes involved in inappropriate splitting. The rate of inappropriate pill splitting was higher in older (over 65 years of age) patients (1.1%, 832/75,387). Eighty-seven percent (1089/1252) of inappropriate prescriptions were prescribed by internists. The rate of inappropriate pill splitting was highest from endocrinologists (3.4%, 429/12,477), nephrologists (1.3%, 81/6,028) and cardiologists (1.3%, 297/23,531). Multivariate logistic regression analysis revealed that the strongest factor associated with individual specific drug of inappropriate splitting was particular physician specialties.

**Conclusion:**

This study provides important insights into the inappropriate prescription of special oral formulation related to pill splitting, and helps to aggregate information that can assist medical professionals in creating processes for reducing inappropriate pill splitting in the future.

## Introduction

Pill splitting is common in oral drug therapy in clinical practice [1]. Pills are frequently split to achieve dose flexibility, facilitate swallowing or reduce medication costs [2], [3]. However, the splitting of certain specific drugs is deemed unsafe, such as extended-release (ER) formulations and enteric-coated (EC) formulations because these specific kinds of formulations could be damaged by pill splitting, resulting in rapid absorption of the drug and a subsequent abrupt rise in blood concentration. Prescribing inappropriate splitting medications may result in unintended clinical outcomes, and are thus considered medication errors [4], [5].

Medication errors may occur at any stage of medication usage, including: prescription, dispensation, and administration. However, prescription is often noted as the stage where most errors occur [6]. Inappropriate dosage forms conducted by medical practitioners represent 11.2 to 20% of total prescription errors, were reported [7], [8]. Splitting oral drugs with ER or EC formulation is one of the common prescribing errors related to dosage form. However, there is little information in the literature about the risk factors of prescription errors associated with inappropriate pill splitting.

The primary purpose of this study was to assess the frequency, and identify the risk factors, associated with inappropriate pill splitting.

## Materials and Methods

### Data Source

This study was conducted in a tertiary care medical center in Taiwan which has more than 2.5 million outpatient visits per year. More than 25,000 ambulatory prescriptions were delivered daily. Data analysis was based on a computerized ambulatory prescription database from January 1^st^ to May 31, 2010. The study variables which were analyzed included: patient gender and age, diagnosis codes (International Classification of Diseases, 9^th^ revision, Clinical Modification, ICD-9-CM), specialty of prescribing physician, prescribed drugs, dose, frequency and route of administration. Patients filled their prescriptions from the same hospital pharmacy.

This study was approved by the institutional review board of Taipei Veterans General Hospital. Since the research posed no more than minimal risk to the participants and involved no procedures, the review board approved that written consent from patients was not required.

### Assessment of Splitting and Divisibility

Drugs were recognized as “special oral formulations” if they were ER or EC formulations that were not originally intended to be split. Drug formulation and the appropriateness of splitting were noted on the instruction leaflets accompanying the medicines. Whenever the prescribed drug was fragmented despite being a special oral formulation, we defined that the drug as split, and regarded it as an inappropriate prescription of pill splitting. The drugs with special oral formulations were supplied consistently and stably over the whole study period.

### Statistical Analysis

All data were linked by the SQL server 2008 (Microsoft Corp., Redmond, WA, USA) and analyzed by SPSS software version 19.0 (SPSS, Inc., Chicago, IL, USA). Descriptive statistics was used to summarize prescription characteristics. For each specific drug with special oral formulation, multivariate logistic regression analysis was used to examine the potential association of independent variables (including: gender, age, and specialty of prescribing physician) with inappropriate pill splitting. The dependent variable was defined as a single prescription with inappropriate pill splitting. We assume that the prescriptions are independent of each other. Patients aged less than 18 years were excluded due to the sample size being too small to yield statistically reliable results of logistic regression analysis. Results are presented as odds ratios and 95% confidence intervals (CIs). A *p* value <0.05 was considered to indicate statistical significance.

## Results

Thirty-five different drugs with special oral formulations in this study are shown in Table 1. During the 5-month study period, 2,162,023 prescriptions were prescribed. A total of 124,300 prescriptions with special oral formulations (5.7% of all prescriptions, 124,300/2,162,023) were prescribed, and 1.0% of these prescriptions (1,252/124,300) were inappropriately split. The characteristics of ambulatory prescriptions with special oral formulations are shown in Table 2.

**Table 1:** Drugs with special oral formulations, selected from TVGH formulary^a^.

Drug formulationand drug class^b^	Drug name (product name)	Potential problems of splitting^c^
**Extended-release formulations**		
Anti-diabetic Agents	Gliclazide MR tab 30 mg (Diamicron®); Glipizide SR tab10 mg (Diabetrol®); Metformin ER tab 500 mg (Ansures®)	May increase risk of hypoglycemia (e.g. gliclazide MR tab), cause gastric irritation (e.g. metformin ER tab), decrease duration of action
Cardiovascular Agents		
Antilipemic Agents	Bezafibrate retard coated tab 200 mg (Bezalip®);Fluvastatin XL tab 80 mg (Lescol®)	May decrease lipid-lowering efficacy
Calcium Channel Blockers	Diltiazem retard tab 90 mg (Cardizem®); Felodipine ERtab 2.5 mg, 5 mg (Plendil®); Nifedipine OROS tab30 mg (Adalat®)	May deliver a toxic dose of the active ingredient, increase risk of hypotension, decrease duration of action
α-Adrenergic Blocking Agents	Alfuzosin XL tab 10 mg (Xatral®); Bunazocin ER tab 3 mg(Detantol®); Doxazosin XL tab 4 mg (Doxaben®)	May increase risk of dizziness, hypotension and reflex tachycardia, decrease duration of action
Central Nervous System Agents		
Analgesics and Antipyretics:NSAIDs	Diclofenac SR tab 75 mg (Meitifen®); Etodolac SR tab600 mg (Eric®, Lacoxa®)	May deliver a toxic dose of the active ingredient, increase gastric irritation
Analgesics and Antipyretics:Opiate Agonists	Morphine SR tab 60 mg (MST®)	May increase risk of drowsiness and respiratory depression, decrease duration of action
Antidepressants	Bupropion SR tab 150 mg (Wellbutrin®)	May increase risk of insomnia, increase risk of hypertension
Anorexigenic Agents	Methylphenidate ER tab 18 mg, 27 mg (Concerta®)	May increase risk of insomnia, increase risk of hypertension and tachycardia
Anxiolytics	Alprazolam XR tab 0.5 mg (Xanax®)	May increase risk of sedation, decrease duration of action
Anti-Parkinson drugs	L-dopa/Benserazide HBS cap 100/25 mg (Madopar®)	May increase risk of nausea, vomiting, and excessive motor activity, decrease duration of action
Electrolytic ReplacementPreparations	Potassium chloride tab 600 mg (Slow-K®)	May cause gastric irritation
Genitourinary Smooth MuscleRelaxants	Tolterodine SR cap 4 mg (Detrusitol®)	May increase risk of somnolence, flushing, and dry mouth, decrease duration of action
Respiratory Tract Agents		
Antihistamines	Loratadine/Pseudoephedrine repetabs tab 5/120 mg (Clarinase®), 10/240 mg (Finska-LP®)	May deliver a toxic dose of the active ingredient, increase risk of dry mouth, nervousness, decrease duration of action
Mucolytic Agents	Ambroxol SR tab 80 mg (Loxol®)	May decrease duration of action
**Enteric-coated formulations**		
Anti-infective Agents: Antivirals	Didanosine EC DR cap 250 mg, 400 mg (Videx®)	May become inactive in the stomach, increase the risk of digestive intolerance
Central Nervous System Agents:Anticonvulsants	Valproate EC tab 200 mg (Depakine®)	May cause gastric irritation
Enzymes	Serratiopeptidase tab 5 mg (Danzen®)	May become inactive in the stomach
Gastrointestinal Agents		
Laxatives	Diphenylmethane EC tab 5 mg (Bisacodyl®)	May cause gastric irritation
Proton pump inhibitors	Rabeprazole tab 20 mg (Pariet®); Pantoprazole tab40 mg (Pantoloc®)	May become inactive in the stomach
Anti-inflammatory agents	Mesalamine tab 400 mg (Asacol®)	May fail to reach terminal ileum and colon of action
Immunosuppressants	Mycophenolic acid tab 180 mg (Myfortic®)	May fail to reach small intestine of action, increase risk of gastrointestinal side effects

Cap, capsule; CR, controlled-release; DR, delayed release; EC, enteric-coated; ER, extended-release; HBS, Hydrodynamically Balanced System; MR, modified release; NSAID, non-steroids anti-inflammatory and antirheumatic products; OROS, Osmotic-controlled Release Oral delivery System; PR, prolonged release; SR, sustained release; tab, tablet; TVGH, Taipei Veterans General Hospital; XL, *extended*-*release;* XR, *extended*-*release*

a Taipei Veterans General Hospital Formulary 2011 Edition

b Drugs were classified by the American Hospital Formulary Service (AHFS) Pharmacologic-Therapeutic Classification System

c Pharmacologic and formulation considerations

**Table 2:** Characteristics of ambulatory prescriptions with special oral formulation.

	Total			<18 yrs			18–64 yrs			>65 yrs		
	n	N	(%)	n	N	(%)	n	N	(%)	n	N	(%)
Variables	1252	124300	(1.0)	14	2133	(0.7)	406	46780	(0.9)	832	75387	(1.1)
**Gender**												
Male	698	76615	(0.9)	8	1493	(0.5)	173	22380	(0.8)	517	52742	(1.0)
Female	554	47685	(1.2)	6	640	(0.9)	233	24400	(1.0)	315	22645	(1.4)
**Prescriber specialty**												
Metabolism & endocrinology	429	12477	(3.4)	–			157	4578	(3.4)	272	7899	(3.4)
Cardiology	297	23531	(1.3)	0	6		81	5838	(1.4)	216	17687	(1.2)
General medicine	149	12590	(1.2)	0	15		27	2767	(1.0)	122	9808	(1.2)
Neurology	88	10435	(0.8)	0	63		24	3282	(0.7)	64	7090	(0.9)
Psychiatry	87	7024	(1.2)	1	1280	(0.1)	55	4265	(1.3)	31	1479	(2.1)
Nephrology	81	6028	(1.3)	0	10		22	2613	(0.8)	59	3405	(1.7)
Surgery	48	8453	(0.6)	1	49	(2.0)	28	4273	(0.7)	19	4131	(0.5)
Others	73	43762	(0.2)	12	710	(1.7)	12	19164	(0.1)	49	23888	(0.2)
**Diagnosis (top 10)**												
Diabetes mellitus	453	14428	(3.1)	–			136	4810	(2.8)	317	9618	(3.3)
Essential hypertension	151	15159	(1.0)	–			40	3854	(1.0)	111	11305	(1.0)
Other forms of chronic ischemic heart disease	103	6311	(1.6)	–			21	1209	(1.7)	82	5102	(1.6)
Hypertensive heart disease	64	7069	(0.9)	–			18	1657	(1.1)	46	5412	(0.8)
Episodic mood disorders	41	3259	(1.3)	0	69		25	2434	(1.0)	16	756	(2.1)
Schizophrenic disorders	22	1100	(2.0)	0	8		22	1015	(2.2)	0	77	
Dementias	20	1517	(1.3)	–			0	53		20	1464	(1.4)
Disorders of lipoid metabolism	19	911	(2.1)	0	1		8	411	(1.9)	11	499	(2.2)
Heart failure	19	825	(2.3)	–			6	115	(5.2)	13	710	(1.8)
Cardiac dysrhythmias	18	1347	(1.3)	0	4		5	284	(1.8)	13	1059	(1.2)

n, number of prescriptions with inappropriate splitting; N, number of prescriptions with special oral formulation.

%, n/N, proportion of prescriptions with inappropriate splitting; –, no prescriptions with special oral formulation.

The rate of inappropriate pill splitting was higher in older (>65 years) patients (1.1%, 832/75,387). Eighty-seven percent of prescriptions (1089/1252) with inappropriate splitting were from internists. The rate of inappropriate pill splitting was highest from endocrinologists (3.4%, 429/12,477), nephrologists (1.3%, 81/6,028) and cardiologists (1.3%, 297/23,531).

Among drugs with special oral formulations, 20 kinds of drugs (57.1%, 20/35) had ever been prescribed to split. For half of these drugs (50.0%, 10/20), the prescribed splitting was unnecessary since they had a corresponding lower strength dose available in the study hospital. The detailed information of prevalence of inappropriate pill splitting for adults is shown in Table 3. Anti-diabetic agents, cardiovascular agents, and central nervous system agents were the most common drug classes involved in inappropriate splitting. The three most frequently prescribed inappropriately split pills were gliclazide modified-release (MR) tab 30 mg (n = 549), fluvastatin extended-release (XL) tab 80 mg (n = 224) and alprazolam ER tab 0.5 mg (n = 117). The top three drug class and physician specialty pairs with the highest rate of inappropriate pill splitting were anti-diabetic agents prescribed by nephrologists (13.2%, 37/280), CNS agents prescribed by cardiologists (11.4%, 56/492) and CNS agents prescribed by nephrologists (10.3%, 4/39).

**Table 3:** The number and proportion of prescriptions with inappropriate splitting, by physician specialty.

	Total		Metabolism		Cardiology		Gen Med		Neurology		Psychiatry		Nephrology		Surgery		Others	
	n	(%)	n	(%)	n	(%)	n	(%)	n	(%)	n	(%)	n	(%)	n	(%)	n	(%)
**All drug items**	**1238**	**(1.0)**	**429**	**(3.4)**	**297**	**(1.3)**	**149**	**(1.2)**	**88**	**(0.8)**	**86**	**(1.5)**	**81**	**(1.3)**	**47**	**(0.6)**	**61**	**(0.1)**
Anti-diabetic Agents	623	(5.8)	347	(6.6)	111	(5.8)	81	(4.9)	10	(1.8)	0		37	(13.2)	7	(2.8)	30	(3.6)
Gliclazide MR tab30 mg (Diamicron®)	549	(6.7)	292	(7.2)	109	(6.7)	74	(6.4)	3	(0.9)	0		36	(14.4)	7	(4.7)	28	(4.7)
Metformin ER tab500 mg (Ansures®)	74	(2.9)	55	(4.8)	2	(0.7)	7	(1.4)	7	(2.9)	0		1	(3.3)	0		2	(0.9)
Cardiovascular Drugs	372	(0.7)	71	(1.1)	127	(0.7)	63	(1.0)	42	(1.0)	2	(0.5)	31	(0.9)	27	(1.4)	9	(0.1)
Fluvastatin XL tab80 mg (Lescol®)	224	(5.3)	45	(5.4)	81	(5.5)	50	(6.1)	16	(3.4)	0		17	(7.5)	15	(10.0)	0	
Felodipine ER tab5 mg (Plendil®)	48	(0.4)	11	(0.7)	10	(0.2)	5	(0.2)	11	(0.7)	2	(0.9)	3	(0.3)	6	(1.1)	0	
Doxazosin XL tab4 mg (Doxaben®)	27	(0.4)	8	(1.4)	9	(0.3)	3	(0.7)	3	(1.2)	0		2	(0.3)	0		2	(0.1)
Diltiazem retard tab90 mg (Cardizem®)	26	(0.7)	4	(1.0)	8	(0.3)	3	(1.9)	0		0		0		6	(4.8)	5	(4.4)
Nifedipine OROS tab30 mg (Adalat®)	18	(0.2)	0		6	(0.1)	0	0	6	(0.7)	0		6	(0.6)	0		0	
Others	29	(0.2)	3	(0.3)	13	(0.4)	2	(0.1)	6	(0.6)	0		3	(0.6)	0		2	(0.0)
Central NervousSystem Agents	210	(0.9)	4	(4.0)	56	(11.4)	5	(1.0)	36	(0.8)	76	(1.7)	4	(10.3)	13	(0.4)	16	(0.2)
Alprazolam XR tab0.5 mg (Xanax®)	117	(6.5)	4	(5.3)	56	(13.4)	0		30	(8.0)	4	(2.2)	0		13	(9.9)	10	(2.0)
Bupropion XL tab150 mg (Wellbutrin®)	59	(3.6)	0		0		5	(11.1)	1	(1.3)	46	(3.2)	4	(80.0)	0		3	(14.3)
Valproate EC tab200 mg (Depakine®)	18	(0.9)	0		0		0		2	(0.3)	13	(1.1)	0		0		3	(1.6)
Others	16	(0.0)	0		0		0		3	(0.0)	13	(0.8)	0		0		0	
Gastrointestinal Drugs	15	(0.1)	4	(0.7)	3	(0.3)	0		0		8	(1.0)	0		0		0	
Others	18	(0.1)	3	(0.9)	0		0		0		0		9	(0.9)	0		2	

Prescriptions for adult (age >18 yrs).

Metabolism, Metabolism & endocrinology; Gen Med, General medicine; n, number of prescriptions with inappropriate splitting.

%, n/N, proportion of prescriptions with inappropriate splitting by specific drug and physician specialty (N: number of prescriptions with special oral formulation by the specific drug and physician specialty in Table S1.).

Multivariate logistic regression analysis revealed that the strongest factor associated with individual specific drug of inappropriate splitting was particular physician specialties. The risk of the top 6 prescribed inappropriate splitting drugs and physician specialties are shown in Table 4. For anti-diabetic agents, endocrinologists were more likely to prescribe inappropriately (gliclazide MR tab 30 mg: OR 8.45, 95%CI 2.69–26.50, compared to neurologists; metformin ER tab 500 mg: OR 7.76, 95%CI 1.87–32.18, compared to endocrinologists). The risk of inappropriate pill splitting was more frequent for surgeons who prescribed gliclazide MR tab 30 mg (OR 5.64, 95%CI 1.44–22.16) than for neurologists, and fluvastatin XL tab 80 mg (OR 4.78, 95%CI 1.51–15.11) than for endocrinologists, also felodipine ER tab 5 mg (OR 5.75, 95%CI 2.07–15.97) than for cardiologists, and more alprazolam extended-release tab 0.5 mg (OR 17.62, 95%CI 2.21–140.41) than for psychiatrists.

**Table 4:** Odds ratios (OR) and 95% confidence interval (95% CI) for association of prescribing inappropriate splitting drugs and physician specialty.

Prescriber Specialty	Gliclazide MR tab 30 mg (Diamicron®)		Metformin ER tab 500 mg (Ansures®)		Fluvastatin XL tab 80 mg (Lescol®)		Felodipine ER tab 5 mg (Plendil®)		Alprazolam XR tab 0.5 mg (Xanax®)		Bupropion XL tab 150 mg (Wellbutrin®)	
	OR^a^	95% CI	OR^a^	95% CI	OR^b^	95% CI	OR^a^	95% CI	OR^c^	95% CI	OR^a^	95% CI
Metabolism & endocrinology	8.45*	(2.69–26.50)	7.76*	(1.87–32.18)	1^d^		3.43*	(1.45–8.11)	11.32	(0.99–129.99)	–	
Cardiology	7.75*	(2.45–24.57)	1^d^		0.83	(0.42–1.67)	1^d^		30.42*	(4.08–227.02)	–	
General medicine	6.99*	(2.19–22.33)	2.25	(0.46–10.94)	2.26*	(1.21–4.22)	1.03	(0.35–3.01)	–		7.92	(0.89–70.69)
Neurology	1^d^		4.42	(0.91–21.51)	–		3.57*	(1.51–8.42)	5.55	(0.67–45.67)	1^d^	
Psychiatry	–		–		–		4.91*	(1.06–22.75)	1^d^		5.80	(0.76–44.05)
Nephrology	19.79*	(6.01–65.18)	4.85	(0.43–55.31)	2.37*	(0.93–−6.04)	1.56	(0.43–5.71)	–		–e	
Surgery	5.64*	(1.44–22.16)	–		4.78*	(1.51–15.11)	5.75*	(2.07–15.97)	17.62*	(2.21–140.41)	–	
Others	5.56*	(1.68–18.46)	1.44	(0.20–10.50)	–		–		0.43	(0.03–6.98)	22.23*	(2.08–237.96)

Multiple logistic regression, Hosmer and Lemeshow goodness-of-fit test: all p>0.05.

a Adjusted by age and sex.

b Female, adjusted by age, sex, sex and physician specialty interactions.

c Age 18–64 yrs, adjusted by age, sex, age and physician specialty interactions.

d The physician specialty which has the lowest proportion of prescriptions with inappropriate splitting.

e Data was excluded due to small sample size (n/N = 4/5).

* p<0.05.

## Discussion

This study revealed the frequency and characteristics of inappropriate prescriptions related to pill splitting. Among all prescribed medications during ambulatory visits, 5.7% of all medications were drugs with special oral formulations. Inappropriate splitting occurred in 1% of drugs with special oral formulations. Fifty-seven percent of drugs with special oral formulations had ever been prescribed to be split. For half of these drugs, the prescribed splitting was unnecessary since they had a corresponding lower strength dose available in the study hospital. Unexpectedly, internists delivered the greatest number of prescriptions with inappropriate pill splitting. The rate of inappropriate pill splitting was highest from endocrinologists (3.4%), to nephrologists (1.3%) and cardiologists (1.3%). This study provides insights into the inappropriate prescriptions with pill splitting. The results demonstrate that efforts to reduce inappropriate tablet splitting are necessary and pressing.

Lewis et al. reported an overall total prescribing error rate of about 7% in a recent systemic review [9]. If our prescription error rate is taken to account with the rate of prescribing error from Lewis et al., inappropriate pill splitting might involve a large proportion of prescribing errors. Based on principles of pharmacodynamics and pharmacokinetics, clinical potential harm from inappropriate splitting (Table 1) is predictable. Lesar et al. showed that the inappropriate use of medication dosage forms is associated with increased risk for adverse clinical events [10]. One case has even been reported in which a crushed extended-release nifedipine tablet delivered, had a fatal outcome [11]. However, the actual harm caused by inappropriate pill splitting requires further research.

Anti-diabetic agents, cardiovascular agents and central nervous system agents were the most common drug classes involved in inappropriate splitting. This finding may indicate that physicians are not fully aware of critical splitting restrictions, which may be due to the deficiency of knowledge regarding special oral formulations. For example, we identified surgery as significantly associated with inappropriate splitting for centain drugs (eg., anti-diabetic agent gliclazide, antilipemic agent fluvastatin, and hypotensive agent felodipine). Surgeons rarely initiate drug therapy outside of their specialty service, and knowledge about these drugs may be poor. In view of these facts, two strategies can be suggested. First, printed hospital formularies should provide detailed prescribing information on tablet splitting, thereby reducing inappropriate splitting of drugs with special oral formulations. Second, a computerized clinical decision-support function within the computerized physician order entry system needs to be implemented, which could be a powerful intervention tool to help avoid inappropriate prescriptions for pill splitting [12].

The reason for internists associated with inappropriate pill splitting may be due to their service specialties. Our data showed that endocrinologists, nephrologists and cardiologists were more likely to be associated with inappropriate splitting among all medical specialties. The probable reason may be that the majority of endocrinologists and nephrologists’ clients are patients with renal insufficiency, such that dose adjustment may be indicated, and pill splitting prescribed [13]. Pharmacies should consider introducing new formulations with lower dosage strength for clinical demand.

Old age was associated with a high risk of inappropriate pill splitting. The changing pathophysiology occurring with the aging process results in complex alterations to the pharmacokinetics and pharmacodynamics of medications [14]. Clinical study has demonstrated that the effectiveness of drugs in geriatrics was substantially metabolized in amounts lower than those standard references doses predicted [15]. Therefore, prescribing the lowest effective doses of medications to older patients may avoid adverse drug events, minimize side effects, and increase compliance. However, this consideration of lowering doses for elders may cause an increased incidence of prescriptions involving tablet splitting, and contribute to a higher rate of inappropriate tablet splitting among older patients.

This study has provided possible factors associated with inappropriate pill splitting for drugs with special oral formulations. The risk factors identified in this study may imply how to develop strategies for preventing medication errors for drugs with special oral formulations. The findings of this study are informative for the assessment and development of medication prescription policy in hospitals. In addition, identification and acknowledgement of risk factors regarding inappropriate pill splitting are needed to incorporate guidelines for medication safety and the educational curriculum of health professionals.

This study has some limitations. First, the study was conducted in a single hospital over a short period of time, which limits the generalizability of our findings. Second, we only assessed the frequency of inappropriate pill splitting by drug formulation without analyzing associated clinical outcomes. Other characteristics of drugs such as score marking, size or shape of pills, and multiple ingredients were not taken into consideration [16]. In addition, patient knowledge and ability to split tablets remain unknown. This could lead to underestimation of the frequency of inappropriate pill splitting. Finally, this cross-sectional study was only designed to identify associated risk factors; it cannot assess causality. However, our data provide insights into the nature of inappropriate prescription of pill splitting in outpatient clinics. Results from this study can provide an important foundation for future research.

## Conclusions

In conclusion, medical prescriptions involving inappropriate pill splitting are not rare in clinical practice. This phenomenon may be due to the lack of knowledge of special oral formulations which cannot be split. This study provides fundamental information about inappropriate pill splitting. However, our study may imply that healthcare providers should take further steps to employ enhanced safety modalities, to prevent or reduce the occurrence of inappropriate prescription pill splitting.

## Supporting Information

Table S1 **Number of prescriptions with special oral formulation by the specific drug and physician specialty.**
(DOCX)
