# Supplementary material for: Association between Physician Specialty and Risk of Prescribing Inappropriate Pill Splitting
Source: PLoS One. 2013 Jul 29;8(7):e70113. doi: 10.1371/journal.pone.0070113 (PMC3726493; doi:10.1371/journal.pone.0070113)
Supplement: Table S1 — Number of prescriptions with special oral formulation by the specific drug and physician specialty. (DOCX) [file pone.0070113.s001.docx]

**Table S1.** Number of prescriptions with special oral formulation by the specific drug and physician specialty

|  | Total | Metabolism | Cardiology | Gen Med | Neurology | Psychiatry | Nephrology | Surgery | Others |
| --- | --- | --- | --- | --- | --- | --- | --- | --- | --- |
| **All prescriptions** | **122167** | **12477** | **23525** | **12575** | **10372** | **5744** | **6018** | **8404** | **43052** |
| Anti-diabetic Agents | 10759 | 5225 | 1916 | 1660 | 559 | 43 | 280 | 250 | 826 |
| Gliclazide MR tab 30 mg (Diamicron^®^) | 8212 | 4068 | 1625 | 1164 | 321 | 41 | 250 | 148 | 595 |
| Metformin ER tab 500 mg (Ansures^®^) | 2547 | 1157 | 291 | 496 | 238 | 2 | 30 | 102 | 231 |
| Cardiovascular Agents | 53367 | 6237 | 19619 | 6534 | 4168 | 400 | 3524 | 2061 | 10824 |
| Fluvastatin XL tab 80 mg (Lescol^®^) | 4242 | 831 | 1483 | 818 | 473 | 3 | 227 | 150 | 257 |
| Felodipine ER tab 5 mg (Plendil^®^) | 13443 | 1571 | 4991 | 2423 | 1521 | 212 | 996 | 564 | 1165 |
| Doxazosin XL tab 4 mg (Doxaben^®^) | 6580 | 570 | 2742 | 451 | 246 | 36 | 678 | 211 | 1646 |
| Diltiazem retard tab 90 mg (Cardizem^®^) | 3727 | 387 | 2824 | 158 | 46 | 11 | 64 | 124 | 113 |
| Nifedipine OROS tab 30 mg (Adalat^®^) | 10345 | 1820 | 4424 | 1154 | 815 | 66 | 1044 | 386 | 636 |
| Others | 15030 | 1058 | 3155 | 1530 | 1067 | 72 | 515 | 626 | 7007 |
| Central Nervous System Agents | 22235 | 101 | 492 | 515 | 4276 | 4395 | 39 | 2948 | 9469 |
| Alprazolam XR tab 0.5 mg (Xanax^®^) | 1792 | 75 | 419 | 89 | 373 | 179 | 17 | 131 | 509 |
| Bupropion XL tab 150 mg (Wellbutrin^®^) | 1622 | 2 | 1 | 45 | 78 | 1448 | 5 | 22 | 21 |
| Valproate EC tab 200 mg (Depakine^®^) | 2067 | 3 | 2 | 31 | 594 | 1183 | 3 | 61 | 190 |
| Others | 16754 | 21 | 70 | 350 | 3231 | 1585 | 14 | 2734 | 8749 |
| Gastrointestinal Drugs | 16665 | 570 | 1123 | 1461 | 852 | 791 | 1124 | 1697 | 9047 |
| Others | 19141 | 344 | 375 | 2405 | 517 | 115 | 1051 | 1448 | 12886 |

Prescriptions for adult (age > 18 yrs).

Metabolism, Metabolism & endocrinology; Gen Med, General medicine.
